# Trends and Challenges of Wearable Multimodal Technologies for Stroke Risk Prediction

**DOI:** 10.3390/s21020460

**Published:** 2021-01-11

**Authors:** Yun-Hsuan Chen, Mohamad Sawan

**Affiliations:** 1 CenBRAIN Lab., School of Engineering, Westlake University, Hangzhou 310024, China; chenyunxuan@westlake.edu.cn; 2 Institute of Advanced Study, Westlake Institute for Advanced Study, Hangzhou 310024, China

**Keywords:** stroke, wearables devices, multimodal technologies, EEG, fNIRS, prediction

## Abstract

We review in this paper the wearable-based technologies intended for real-time monitoring of stroke-related physiological parameters. These measurements are undertaken to prevent death and disability due to stroke. We compare the various characteristics, such as weight, accessibility, frequency of use, data continuity, and response time of these wearables. It was found that the most user-friendly wearables can have limitations in reporting high-precision prediction outcomes. Therefore, we report also the trend of integrating these wearables into the internet of things (IoT) and combining electronic health records (EHRs) and machine learning (ML) algorithms to establish a stroke risk prediction system. Due to different characteristics, such as accessibility, time, and spatial resolution of various wearable-based technologies, strategies of applying different types of wearables to maximize the efficacy of stroke risk prediction are also reported. In addition, based on the various applications of multimodal electroencephalography–functional near-infrared spectroscopy (EEG–fNIRS) on stroke patients, the perspective of using this technique to improve the prediction performance is elaborated. Expected prediction has to be dynamically delivered with high-precision outcomes. There is a need for stroke risk stratification and management to reduce the resulting social and economic burden.

## 1. Introduction

Stroke ranks as one of the top first leading causes of death and disability worldwide [1,2], particularly for the most populous countries in Asia, Europe, and North America. It is the number one cause of death and disability in China [3], and it is the 2nd and 4th leading cause of death in Germany and United States, respectively [1]. However, 80% of strokes are preventable if risk factors can be controlled [4]. Hypertension is one of the major risk factors for both ischemic and hemorrhage strokes [5]. Also, hyperglycemia, hyperlipidemia, obesity, diabetes, atrial fibrillation, smoking, heavy drinking, sedentary lifestyle, and unhealthy diet are among the well-known risk factors to control [1]. Many available guidelines describe “population-wide” and “high-risk” strategies intended for stroke prevention [6,7,8]. For population-wide strategies, various scoring systems are developed to evaluate the risk of stroke according to specific risk factors, such as health condition, lifestyle, behaviors, and family history of diseases [9]. Actions are proposed to people to control risk factors, such as changing lifestyle behaviors or taking medicine, and the variation in the risk of stroke is tracked by evaluating the risk of stroke annually. However, those who are identified to have a high risk of stroke are directed to high-risk prevention strategies [10]. The procedures contain sub-clinical examinations, such as carotid ultrasound and transcranial doppler (TCD) which can better characterize the cerebrovascular function and analyze the consequences of reduced function on the risk of stroke [11]. In addition, according to various levels and causes of risk factors, drugs, surgeries, or regular follow-up examinations are carried out to prevent stroke.

The above-described strategies to prevent stroke are beneficial but present several limitations. First, the accessibility of survey or sub-clinical examinations can be low due to geographical or resource limitations. Second, the self-reported personal and family health conditions can be subjective or difficult to quantify. Third, the physiological conditions in the strategy for stroke prevention are not characterized or evaluated in real-time, which cannot reflect the instant variation of the health condition. Fourth, the collected physiological parameters can only indirectly reflect the risk of stroke. For example, a blood vessel is blocked by a blood clot that cannot be identified by an instant blood pressure value measured by a wrist or an arm blood pressure cuff. Fifth, the preventive reactions can only be carried out on those being involved in a stroke prevention project or system including several follow-up visits. Moreover, a person’s will to take sub-clinical examinations or participate in follow-up visits and examinations regularly is difficult to control. Last, the risk of stroke can be estimated, but the possible onset time of stroke cannot be determined.

**Figure 1:**
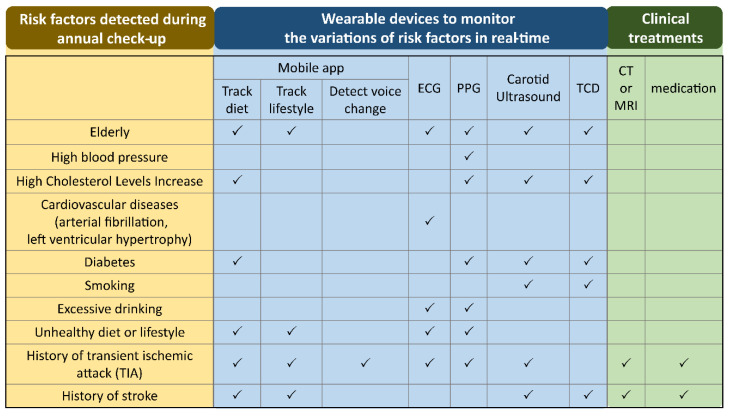
The wearable devices to monitor the variations of physiological parameters in real-time when a person is diagnosed with certain risk factors during an annual check-up. ECG: electrocardiogram; PPG: photoplethysmography; TCD: transcranial Doppler; CT: computed tomography; MRI: magnetic resonance imaging.

The emerging wearable devices intended to monitor the physiological parameters, and the growth of machine learning applied to predict diseases, are promising solutions to prevent stroke and eventually predict stroke risk [12]. In fact, wearable devices are easy to use, allowing monitoring of the variation of vital signs continuously without impeding the normal life of people. These devices can be used by people living in various areas (rural and urban) where medical resources and infrastructure can be insufficient. The wearable devices used for real-time monitoring of physiological parameters when a person is diagnosed with certain risk factors during annual checkups are shown in Figure 1. Regarding monitoring the cholesterol level using a mobile app, although around two-thirds of cholesterol is synthesized by the liver and only one-third of cholesterol level depends on the diet, the cholesterol influenced by food intake is the part which can be controlled and monitored by wearable devices. Regarding excessive drinking, it has been shown that alcohol consumption is associated with changes in ECG, such as heart rate, heart rate variability (HRV), P-wave, and QTc prolongation [13,14]. The recorded data listed in Table 1 can be instantly analyzed then compared with the historical health records of a database containing a person’s personal and family health records. Regarding machine learning techniques, they are applied to analyze the risk of stroke according to the instantly recorded physiological parameters and a person’s electronic health records (EHRs) [15,16]. A prediction system may evaluate the risk early enough in time to reduce the stress of a person, and increase the efficiency of medical interventions.

We review in this manuscript trends and challenges of wearable multimodal technologies for stroke risk prediction. The remaining sections include an introduction of the materials and methods of collecting the materials for this review in Section 2. Section 3 describes the main wearable technologies for monitoring stroke-related physiological parameters, and the results proving the association between the recorded vital signs and the risk of stroke. The advantages and drawbacks of these wearable technologies and the potential internet-of-things (IoT) systems for stroke risk prediction are the objects of Section 4. In Section 5, the establishment of a risk prediction system, the strategies for using wearable technologies, and the importance of using multimodal electroencephalography-functional near-infrared spectroscopy (EEG-fNIRS) devices for stroke risk prediction are presented. Conclusions are the subject of the last section.

## 2. Materials and Methods

Web of Science and Google Scholar databases are applied for searching the papers related to this review. The materials for Section 1 are searched by the key words “guidelines”, “risk factors”, and “stroke”. The risk factors which can be measured using wearable technologies are selected as the materials for Section 3. The materials for Section 3 are searched by the key words “stroke” or “ischemic stroke” or “neurovascular diseases”, “(risk) prediction” or “predict” and various types of wearable devices which can be used to monitor the risk factors. Key words “internet of things (IoT)” and “stroke” are applied to search the materials for Section 4. For the materials of Section 5, the key words used are “machine learning (ML)” or “deep learning”, “electronic health records (EHRs)”, “multimodal”, “stroke” and “risk prediction” or “predict”.

Papers discussing stroke risk prediction using methods other than wearable technologies, such as laboratory tests (blood, urine, etc.), are not considered in this review. In addition, papers discussing the prediction of any situation on patients who already have stroke are excluded. For example, papers aim to predict the outcome of stroke patients or to predict the risk of secondary stroke are not included in this review.

## 3. Wearable Devices for Stroke Risk Prediction

**Figure 2:**
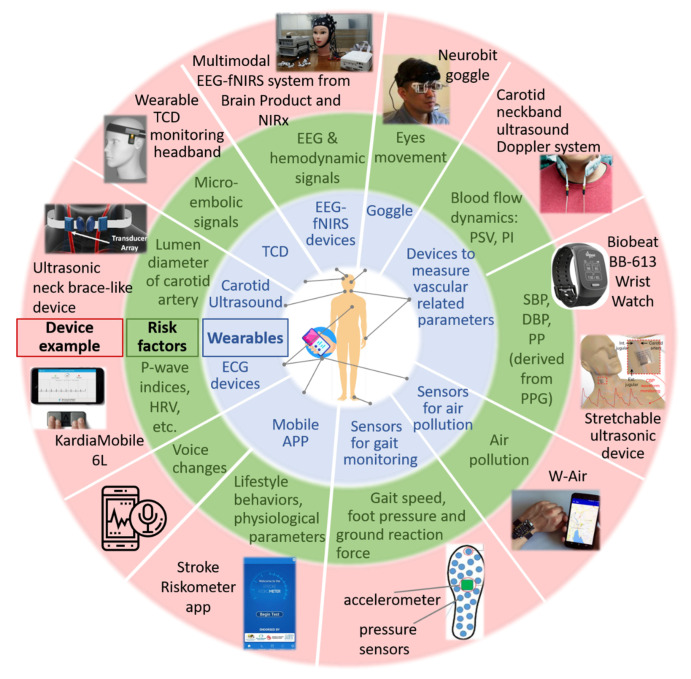
Wearable devices and mobile applications for stroke risk prediction. The wearable devices include sensors for air pollution, devices for measuring vascular-related parameters, carotid ultrasound and Transcranial Doppler (TCD), a gait monitoring system consisting of an accelerometer and pressure sensors, goggles for monitoring eye movements and multimodal Electroencephalography (EEG), and functional near-infrared spectroscopy (fNIRS) devices for monitoring cerebral electrical and hemodynamic activities. DBP: diastolic blood pressure, ECG: electrocardiogram; EEG: electroencephalography; fNIRS: functional near-infrared spectroscopy; HRV: heart rate variability; PI: pulsatility index; PP: pulse pressure; PPG: photoplethysmography; PSV: peak systolic velocity; SBP: systolic blood pressure; TCD: transcranial Doppler.

Available wearable technologies to measure the physiological parameters associated with the risk of stroke are summarized in Figure 2. Besides, mobile applications to evaluate one’s risk of stroke are included. Also, the risk factors resulting from these technologies are discussed, and the advantages and drawbacks of various technologies are described below.

### 3.1. Questionnaires and Scoring Systems via Mobile Applications

Various questionnaires and scoring systems are available to evaluate one’s risk of stroke according to the self-reported personal health condition and lifestyle habits and behaviors [17]. Since the popularity of the smartphone is high, conveying the questionnaires or scoring systems via mobile applications (mobile app) increases the accessibility of this population-wide strategy for stroke risk stratification [18]. The Stroke Riskometer is a mobile app endorsed by the World Stroke Organization to evaluates one’s risk of stroke over the next 5 to 10 years based on 20 questions. In addition, it provides suggestions to lower the risk of stroke [8,19]. Another stroke risk system is specifically developed for the Chinese population to predict 10-year and lifetime stroke risk (Table 1) [20].

These questionnaires are developed based on Framingham risk score, which is the oldest scoring system developed for stroke risk prediction [21]. During evolution of the past 20 more years, around 10 other scoring systems have been developed based on the same Framingham risk score [22,23]. These scoring systems are mainly applied on patients with atrial fibrillation (AF) to stratify their risk of stroke. AF is the most common disorder of heart rhythm, which accounts for at least 20% of all types of stroke [24]. AF means rapid and irregular beating of the atrial chambers of the heart resulting in abnormal heart rhythm. Blood can be stagnated and thrombus can be formed within the left atrial appendage due to the dysrhythmia. This can lead to cardioembolic stroke. The risk of stroke of patients with non-valvular AF is five times higher than those without AF [25]. These scoring systems help to stratify patients with AF to benefit most from anticoagulation, which is reported to reduce more than two-thirds of the incidence of stroke [25].

The scoring systems available as mobile apps are easy to access and intuitively interpret results. However, there are still limitations for these scoring apps. First, people need to know their common physiological parameters, such as systolic blood pressure (SBP), diastolic blood pressure (DBP), high-density lipoprotein (HDL) cholesterol, and low-density lipoprotein (LDL) cholesterol when conducting the questionnaires. Second, the systems do not allow real-time updates of the physiological parameters. Third, a scoring system may be not validated for people with various ethics or at various regions. Fourth, these assessments cannot suggest the risk of stroke in the near future, such as coming weeks or months.

To improve current scoring systems, mobile apps can be connected to wearable devices which record the real-time physiological parameters. The processing algorithms secure better assessment when using the variation of the imported physiological parameters. In addition, customizing the apps for individuals can increase the overall outcome of scoring systems.

### 3.2. Sensor for Air Pollution Embedded in Smart Phone

The association of air pollutants, such as airborne particulate matters up to 2.5 µm in diameter (PM_2.5_), and various toxic gases with stroke have been evaluated this decade [26,27]. It is proved that short-term exposure of PM_2.5_ and the toxic gases are associated with an increase in hospitalization due to ischemic stroke in China [28]. In fact, up to 29% of the risk of stroke is attributable to air pollution [4]. Worryingly, unlike most other modifiable risk factors, air pollution is unavoidable. Domestic and worldwide policies to lessen the impact of air pollution on risk of stroke are urgent needs.

An air pollution detector can validate both the daily concentration and the duration of such pollution. For example, W-Air is a platform embedded in a wristband for air pollution monitoring [29]. It allows measuring toxic gases of the environment without the influences of the gases emitted by the users. However, W-Air is not able to monitor PM_2.5_. A miniaturized mass sensing based on a micro-electro-mechanical system (MEMS) structure is developed to monitor PM_2.5_ [30]. However, the main criterion of air pollution sensor for stroke risk prevention is to continuously monitor both PM_2.5_ and toxic gases, and the measured values should not be influenced by other scents emitted by the user. Also, the wearable device is better integrated with a mobile phone to avoid wearing extra devices [31]. Besides improving the sensor, increasing the public awareness of the risk of stroke resulting from air pollution represents a key step to gain the effectiveness of these sensors.

### 3.3. Devices for ECG Monitoring

Patients with atrial fibrillation, determined by the irregularity of the pulse rate, have a five-times higher risk of stroke than those without AF as discussed in Section 3.1. Among those with clinical AF, one third of them have subclinical AF, which can also result in thromboembolic occurrence and leads to ischemic stroke [32]. People with subclinical AF have no obvious symptoms and often cannot be detected using a conventional short-term Electrocardiogram (ECG) exam, resulting in less attention to their risk of stroke. With the emergence of 24-h portable ECG or cardiac implantable electronic devices (CIEDs), such as a pacemaker, subclinical AF and device-detected AF can be detected, increasing the accessibility of the risk factors of stroke [33,34,35]. Moreover, with the dramatic increase in the accessibility to ambulatory ECG via wearable devices, other cardiac risk factors related to stroke can be monitored continuously and analyzed in real-time [36].

Apple Watch (Apple Inc., Cupertino, CA, USA) series 4 extracts heart rate from recorded photoplethysmography (PPG) [37,38]. The incorporated algorithm, which is the first to obtain Food and Drug Administration (FDA) clearance to determine AF, derives the pulse rate from the peak to peak interval of PPG pulsations [39]. However, pulse rate irregularity is not the only character of AF, other irregular electrical activity of ECG recording can be used to identify AF. Therefore, wearable devices that enable ECG monitoring benefit more on stroke risk prevention. Thus, another ECG device called KardiaMobile 6L from AliveCor, a smartphone attachment enabling 6-lead ECG recoding, is presented [37]. It is also an FDA cleared device for detection of AF. The integrated KardiaAI platform can distinguish AF between normal sinus rhythm based on the recorded ECG signals.

Both algorithms of the discussed devices are designed to determine the presence of atrial fibrillation. However, it has been shown that AF is not always necessary for the formation of thrombus and the occurrence of embolization [40]. Abnormal atrial structure and function can also result in thrombosis and then increase the risk of stroke, even in people without AF [41]. These abnormalities found in P-wave indices, Q-wave, QRS/QT duration, other waveform angles and slopes, and HRV are associated with risk of stroke [42,43,44].

The wearable devices enabling the monitoring of these factors are often in the form of a chest patch, chest strap, or garment. The usability and user comfort of these devices lead to a trade-off with the number of ECG channels. The limited recording lead reduces the applicability of these devices on stroke risk prediction compared to conventional 12-lead ECG. Besides the challenge of balancing the form factor and applicability of wearable ECG devices, there are other limitations. The first limitation is that the ECG measurement is recommended to be conducted when resting, since the motion artifact disturbs the recorded PPG or ECG signals. Second, the algorithms developed and released nowadays are restricted to irregularity of pulse rate due to the complexity of real-time analysis of other cardio abnormalities related to risk of stroke. Third, consultation of professionals for further inspection and diagnosis when abnormal ECG signals are detected is still needed. Fourth, the recorded results can be biased when lack of active and continuous monitoring.

To increase the impact of ECG devices on stroke risk prediction, developing user friendly devices to record meaningful signals for stroke risk analysis is the first step. Secondly there is a need to increase their accessibility in underdeveloped countries, where prevalence of stroke is often higher. The third step is to develop algorithms with high accuracy and specificity to reduce unnecessary anxiety or further testing. Fourth, combine EHRs with the recorded ECG to customize the algorithms resulting a dynamic prediction system. Fifth, increase the awareness of stroke risk prevention using wearable devices and encourage the users to actively take actions when receiving warming signals of abnormal ECG.

### 3.4. Devices for Vascular Related Risk Factors Monitoring

Since stroke is a neurovascular disease, the abnormalities of vascular related risk factors strongly associate to the risk of stroke. In this section, devices enabling continuous monitoring of blood pressure, pulse pressure, arterial stiffness, and blood flow dynamics for stroke risk prediction are introduced.

#### 3.4.1. Blood Pressure Monitoring

Hypertension, counting for up to 50% of cases, is the leading cause among all risk factors of stroke [45]. It causes changes in cerebrovascular structure resulting in the reduction of inner diameter or atherosclerosis of blood vessels. The released fragments or debris of atherosclerotic plaques flowing in the blood vessels can cause stroke [45]. Therefore, tracking the variation of blood pressure (BP) suggests the changes of risk of stroke [46].

Sphygmomanometers are widely accessible for BP monitoring. However, these cuff-based and cumbersome devices hardly provide continuous monitoring [47]. With the booming of various types of non-invasive, portable, and cuffless sensors or sensor systems, continuous monitoring of blood pressure and parameters related to vascular properties is feasible [47,48]. PPG embedded in a smartwatch or a wristband (Biobeat BB-613) is a promising optical technique to measure blood volume changes per pulse, which can be used to determine BP using various algorithms [49,50]. Besides, a wearable stretchable ultrasonic device placed on carotid artery is proposed to continuously measure the central blood pressure waveform, which shows higher relevance to cardiovascular activities comparing to the superficial peripheral BP measured using PPG [51].

These ambulatory recording devices facilitate continuous monitoring of BP in daily life which correlates more with the occurrence of stroke than that measured in clinics [52]. In addition, it is proved that the BP monitored during evening or while sleeping predicts the risk of stroke more precisely than that monitored at any other time during a day [53,54,55,56]. Besides BP, pulse pressure (PP), and the difference between systolic blood pressure (SBP) and diastolic blood pressure (DBP) are other risk factors which can be derived from recorded BP. It is reported that the risk of stroke incidence can be raised by a 10 mmHg increase in PP [57].

Besides the parameters determined from the measured BP, the consequences of BP can be associated to risk of stroke. For example, longstanding hypertension resulting in the accumulation of molecules on the wall of arteries thus increases the stiffness of these arteries [58]. Therefore, arterial stiffness which can be characterized by the second derivative wave of PPG is another vascular related risk factor of stroke [59].

#### 3.4.2. Blood Flow Dynamics Monitored by Doppler Ultrasonographic System

Higher degree of carotid stenosis implies higher risk of stroke [60,61]. The conventional approach to access the degree of carotid stenosis and characterize the carotid plaque is carotid ultrasound, which is introduced in Section 3.5. However, the conventional hand-hold carotid ultrasound is bulky and needs to be conducted by professionals. An ultrasound Doppler system embedded in a carotid neckband is developed for continuous blood flow velocities monitoring. The neckband equipped two ultrasound transducers enables monitoring of left and right arteries. The peak systolic velocity (PSV) of the recorded Doppler waveform suggests the severity of carotid stenosis [62]. Another character of carotid blood flow relates to stroke is carotid pulsatility index (PI) [63].

However, there are limitations of these growing wearables for vascular related risk factors monitoring. First, to obtain reliable signals in daily activities, designs of the devices and algorithms for signal processing need to be improved to compensate the interreferences from motions. Second, the BP derived from current algorithms varies 5–10 mmHg from that recorded from conventional standard techniques. This implies that probably only the variation of derived BP can be valuable for stroke risk prediction. Third, the user can miss the optimal location to place the device and the results can vary due to various locations of the device.

### 3.5. Devices for Carotid Plaque Characterization and Cerebral Microembolization Monitoring

Non-invasive imaging techniques, such as computed tomography (CT) and magnetic resonance imaging (MRI) are the gold standards to characterize the structure of blood vessels (accumulation of plaques, development of atherosclerosis and reduction of lumen diameter) and detect blood flow speed. In this section, two more compact and user-friendly imaging techniques compared with CT and MRI are introduced: ultrasound for carotid plaque monitoring and TCD ultrasonography for embolic signal detection.

#### 3.5.1. Carotid Ultrasound for Carotid Plaques Characterization

Up to 20% of ischemic strokes is caused by the atherosclerosis in a carotid artery [64]. It has been considered that the narrowing and hardening of the carotid artery caused by the accumulation of atherosclerosis plaques limits the blood flow resulting in the increment of stroke risk. Therefore, ultrasound is applied to evaluate the carotid intima-media thickness (CIMT) for classifying the degree of carotid stenosis caused by carotid atherosclerosis [65,66]. However, conventional carotid ultrasound needs to be performed by a professional and the result needs to be interpreted by a physician. Easy to use and cost-effectiveness devices for carotid artery imaging increase the usability of ultrasound technique for stroke risk prediction.

A wearable ultrasonic neck brace-like device is developed for convenient lumen diameter, which associates with CIMT, monitoring without the limitation of time and location [67]. The vertically aligned transducer array of the device avoids the measurement error that often happens during hand operation. Nevertheless, the locations of ultrasound transducers are user controlled and not easy to modify as handhold transducers, which can result in incomprehensive results.

Although CIMT is a common risk factor includes in the scoring systems for stroke risk prediction, in recent years research investigations suggest that the parameters of the instability of carotid plaques contribute more on stroke risk [68,69,70]. The characterized parameters of carotid plaques include the morphology, the composition, the biomechanical force, and other properties [11,71,72,73]. These parameters help to identify the vulnerable plaques, which might rupture and then protrude the lumen resulting in serious stenosis or block completely the blood flow at the stenotic area. Moreover, the debris from the ruptured plaque can move to small vessels in the brain and impede the hemodynamic status resulting in embolic stroke, which accounts for approximately 18–25% of ischemic stroke [71]. The morphologies of carotid plaque need to be carefully scanned using both transverse and sagittal projections enabling overall images for instability validation. Unfortunately, there are no wearable ultrasound devices providing morphology characterization of the carotid plaques nowadays due to the limited adjustment angles of ultrasound transducers embedded in a wearable device. With the increase in the flexibility of the ultrasound transducer probes, a self-guided positioning system, and optimized algorithms for images analysis, a smart wearable ultrasound device for plaque quantification can be helpful for stroke risk prediction.

#### 3.5.2. TCD for Cerebral Microembolization Monitoring

TCD ultrasonography is a specialized ultrasound technique to measure cerebral blood flow (CBF) and cerebrovascular hemodynamics resulted from various physiological as well as pathological states [74]. TCD detects micro-embolic signals (MES) in cerebral vessels in real-time. The frequency of MES during a 1-h TCD monitoring at entry point or at 6, 12, 18 months can predict risk of stroke over the subsequent 6-month period [75,76,77]. Other hemodynamic parameters, such as mean flow velocity, peak systolic and end diastolic flow velocities, and vasomotor reactivity measured using TCD are potential risk factors for stroke prediction [78]. It is found that the increasing mean flow velocity measured from middle cerebral artery implies higher stroke risk.

The limitation of TDC is that the high-quality signals are hardly obtained since they are prone to motion artifacts. Besides, any physiologic changes impact the blood flow velocity, so the measured velocity change must take the related variables into account [74,79]. In addition, experienced operators are highly dependent to obtain high quality TCD signals using optimal acoustic window and probe orientation [80]. Therefore, limited prototypes of wearable TCD for cerebrovascular parameters monitoring are proposed to the best of our knowledge [81,82]. The TCD transducer is placed on the transtemporal location using a pair of glasses or a headband limiting the flexibility of adjusting the transducer probe freely to other optimal position for imaging. In addition, the algorithm needs to be improved for better acoustic window finding methods. Besides the improvement of TCD transducer positioning, the algorithm of automated embolic signals detection technique is needed to increase the benefit of TCD on stroke risk prediction.

### 3.6. Gait and Motion Monitoring

When stroke cases occur, the patients experience muscle-related difficulties, such as blindness or blurred vision, unclear voice, walking or maintaining balance problem, difficulty moving some muscles, and weakness in the limbs or muscles.

A gait monitoring system including an accelerometer and pressure sensors to record gait speed, foot pressure, and ground reaction force is proposed for stroke risk prediction [83,84]. Another promising system for stroke risk prediction is developed to perform gait analysis without specific alignment motions. The algorithm along with the system distinguishes the level of disability of stroke survivors by analyzing the asymmetry of gait parameters measured from lower limbs [85]. Another study statistically analyzes gait nonlinear patterns to distinguish healthy young subjects (23–29 years old), healthy elderly subjects (71–77 years old), and patients with Parkinson’s disease. The complexity measures, walking stride time series, can be potential parameters to predict the risk of stroke [86]. The machine learning technique is used to extract the features and perform classification. In addition, wearable sensors for motion and surface electromyography (EMG) monitoring are widely used to evaluate the rehabilitation of muscle function and motion ability on stroke patients [87]. The EMG signals indicating the transformation of impaired to normal condition of muscles can be applied as features to predict the occurrence of stroke when a part of muscle function is weakened. A wireless body area network composed of multiple sensor nodes and machine learning algorithms are applied to analyze the motion performed by human body. This is a promising system for stroke risk prediction [88].

Besides the loss of muscle function of the limbs, the ocular muscles often become uncontrolled in stroke patients. Therefore, a goggle which combines a pair of glasses and a motion detecting camera is designed by Neurobit for early stroke detection or prediction [89]. By recording the eye movements when a subject is performing the assigned tasks, the algorithm of the system stratifies the risk of stroke. Moreover, people with stroke can experience difficulties in speaking clearly and fluently. A mobile application program is developed to predict risk of stroke by analyzing the users’ voice input [90].

### 3.7. Devices for EEG Monitoring

The above presented devices, except TCD, monitor the physiological parameters indirectly related to cerebrovascular itself. Wearable devices enabling brain activities monitoring should increase the prediction outcome. Since the changes of cerebral blood flow impact the activities of neurons, EEG signals contain stroke risk predictors. The alpha waves decline when the CBF decreases. The theta and delta waves appear when the CBF further decreases [91,92,93]. Besides the sub bands analysis of EEG, the local brain symmetry index (BSI), relevant delta power, and relative local delta to alpha ratio (DAR) calculated from the recorded EEG signals are used as indices for detecting stroke. More examples of applying EEG monitoring on stroke patients from prevention to rehabilitation stages are discussed in Section 5.1.

### 3.8. fNIRS Devices for Hemodynamic Signals Monitoring

Neural electrical activities recorded using EEG are associated with the cerebral hemodynamic, which can be monitored using functional near-infrared spectroscopy (fNIRS). This neurovascular coupling means that when neuron activity arouses, CBF around the area increases to supply more oxygen for neuron activity. fNIRS measures the concentration change of oxygenated hemoglobin ([HbOxy]) and that of deoxygenated hemoglobin ([HbDeoxy]) [94,95]. Together with the derived parameters, HbT (the combination of the previous two parameters) and rSO_2_ (regional cerebral tissue oxygenation), these values are used to evaluate the hemodynamic states of stroke patients. More examples of applying EEG monitoring on stroke patients from prevention to rehabilitation stages are discussed in Section 5.1.

## 4. Comparison and Combination of Various Techniques

In previous Section 3, we introduced various mobile-based and wearable devices facilitating monitoring of stroke risk factors. Each one of these techniques is based on different measuring principles and the measured physiological parameters are used to evaluate the abnormalities occurs from different parts of the body, such as cardiovascular and neurovascular systems (Table 2). The comparison of these technologies and the possibility of integrating them in an IoT platform for stroke risk prediction are discussed below.

Table 3 compares these techniques based on various requirements of wearable devices, such as cost, weight, accessibility, response time, user friendliness, etc. The size and weight of the devices are associate with their cost, accessibility, and frequency of use. The more compact of the devices, with lighter forms and with less skin area covered when worn, result in higher user comfort. The user-friendly devices are mobile phones (with a scoring questionnaire or an air pollution sensor on it) and an insole gait monitoring system. The ECG chest patches, and PPG wristbands come after. The TCD headbands, and EEG-fNIRS caps rank after. CT and MRI are the bulkiest among all these devices.

The accessibility of most devices is proportional to their cost and weight, except for carotid ultrasound neckbands and TCD headbands. The wearable devices of these two technologies are not as mature as other devices. Most devices have a short preparation and response time which assures data continuity during real-time monitoring. However, answering the questionnaires on the mobile phones can take up to five minutes, slowing down their response time. The preparation, measurement, and interpretation time of CT and MRI can take at least an hour, which are the longest among all devices. Regarding the recorded data, ECG and EEG provide high time resolution data, but it is difficult to specify the locations of lesions when abnormal signals are detected. Compared with the large number of the sensors of EEG or fNIRS devices (EEG can have up to 256 EEG electrodes) which can provide information on the overall brain area, the limited number of the sensors of PPG devices, carotid ultrasound neckbands, and TCD headbands restricts the field of view provided by these devices.

To increase the accuracy of the interpretation of risk of stroke, several techniques in an IoT network are combined, facilitating the collection of various physiological parameters. This broadens the applications for various diseases and enhances the prediction ability of a prediction system [96]. One system is composed of watches for blood glucose, blood pressure, and heart rate monitoring [97].

Another system includes a wristband and two Doppler detectors to measure blood pressure and the blood flow of the internal carotid artery and cerebral major artery, respectively [98]. Still another system includes an ECG as well as foot pressure sensors and an accelerometer placed into the insole to measure the gait acceleration, foot pressure, ground reaction force, and other gait signals mentioned in Section 3.6 [83,84]. Together with the signal processing and decision-making algorithm, the results of stroke risk stratification are sent to the users as well as the clinical staff. However, these systems all are not applied to stratify the risk of stroke of those with no stroke before. They are applied either to predict the reoccurrence of stroke on patients had transient ischemic attack (TIA) or used to classified patients with stroke and healthy subject.

Another IoT system is proposed to predict wake-up stroke, which happens during night-time sleep or within 30 min of awakening [92]. The system includes various wearable sensors, such as EEG, ECG, EMG, electrooculography (EOG), PPG, and polysomnography, for stroke-related physiological parameters measurements. In addition, the modifiable and non-modifiable risk factors and EHRs are also included in their prediction system. To precisely predict stroke, using the tremendous amount of data collected by the wearable sensors and other resources, machine learning (ML) approaches, discussed in Section 5, are needed. To the best of our knowledge, there is no well-developed IoT system including multimodal sensors for stroke risk prediction published in journal papers or announced in public. All the systems discussed in this section are only in the proof-of-concept stage.

## 5. Perspectives of Stroke Prediction

The risk factors measured using wearable devices help to understand one’s health condition in time. Meanwhile, the histories of diseases, treatments, medications, and other personal medical data available in EHRs also contain useful information for stroke risk prediction [99]. With the abundant data of real-time recorded physiological parameters and of the EHRs, ML techniques to analysis the association and rank the importance of the risk factors to the risk of stroke are needed [12,100]. The most popular ML model applied for stroke risk prediction is support vector machine (SVM). In recent years, deep neural networks (DNN) gained attention [101,102]. These DNN models are constituted by several layers of neural networks extending the ability of these model to study the relationship between input features and the output through the hidden layers [103]. The important features selected by the ML models for stroke risk prediction are age, history, or family history of hypertension or heart disease, smoking and diabetes, which match with the results of clinical studies. In addition, some ML models suggest that creatinine, glucose, and Hemoglobin A1c (the average blood sugar level for the past two to three months) are risk factors.

It is worthy to learn that a convolutional neural network (CNN) model integrates both EHRs and 24-h data recorded from wearables, such as ECG, EMG, blood pressure, and heart rate for stroke risk prediction, which is not common in other prediction models [15]. The streaming data from wearables is converted into graph during CNN model training. This multi-modal data processing is a trend of future stroke risk prediction since real-time recorded physiological parameters increase the prediction power of the prediction systems comparing those consider only EHRs [104].

Although building an IoT network consisting of many wearables is an approach to increase the prediction power of the prediction models, an ideal stroke risk prediction system should classify various stages of applying different wearables and further implement multi-modal monitoring to maximize the efficiency of the overall system. Efficient strategies of applying the wearables for stroke risk prediction are proposed in Section 5.1. Then, the future perspectives of applying multimodal EEG-fNIRS for stroke risk prediction is discussed in Section 5.2. Last, the challenges, limitations, and possible solutions of applying EEG-fNIRS for brain activity monitoring is discussed in Section 5.3.

### 5.1. Strategies of Using Wearable Technologies for Stroke Risk Prediction

**Figure 3:**
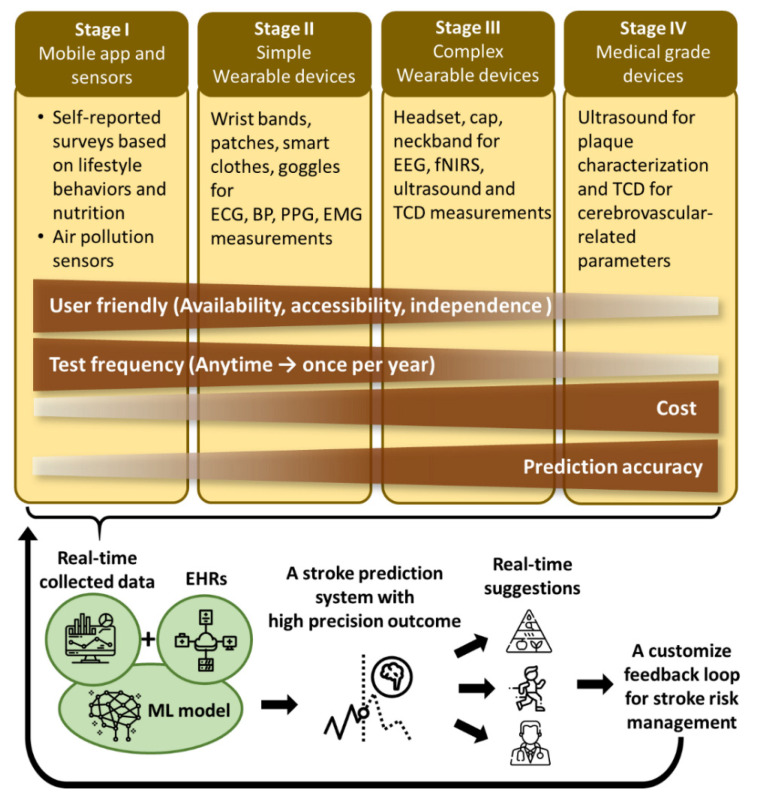
Various techniques introduced in this manuscript are suggested to be applied at different stages for stroke risk prediction. BP: blood pressure; ECG: electrocardiogram; EEG: electroencephalography; EHRs: electronic health records; EMG: electromyography; fNIRS: functional near-infrared spectroscopy; ML: Machine learning; PPG: photoplethysmography; TCD: transcranial Doppler.

Due to various characteristics of the wearable technologies compared in Table 3, classifying the stages of using these wearables increase the efficiency for stroke risk prediction (Figure 3). At the beginning when one gets in touch with a stroke risk prediction system, the number of wearables to apply depends on the comfortability. Then, the types of wearables can be adjusted based on the dynamically predictive stroke risk and real-time suggestions for stroke risk management to avoid the waste of resources and the burden on mind and body. The time of use of the wearables at each stage is described as follows.

Stage I: A self-reported questionnaire regarding the lifestyle behaviors and intake nutrition can be conducted anytime daily. Some inputs of the questionnaire can be automatically obtained using additional function of a mobile phone, such as using accelerometers to count steps and mobile application to record daily diet. This can simplify the questionnaire and save time.

Stage II: Simple wearable devices—such as smart watch, wristband, patch, smart cloth, and goggles—monitor cardio-, vascular-, and muscle- related parameters continuously. These light and user-friendly devices can be used independently without impeding daily activity.

Stage III: Complex wearable device, such as cap, headset, or neckband, using electrical and optical techniques for brain activity and carotid artery status monitoring. These techniques include EEG, fNIRS, ultrasound and TCD, which often cost more than a simple wearable device in Stage II. In addition, these devices are bulkier and more complicated than the simple wearable devices, resulting in demotivation. With these acceptable disadvantages, they are still useful tools for monitoring the risk factors of stroke with an extended time frequency, such as once a week.

Stage IV: although wearable carotid ultrasound and TCD devices are applied for stroke risk prediction at Stage III, more precise results of these measurements can only be obtained using conventional medical grade devices operated by trained professionals. The images of the overall region of interest can be scanned giving comprehensive results. Since these medical grade instruments are only available in the hospitals, these measurements are often carried out only in annual physical exams. Those with known disease histories resulting in higher risk of stroke, such as carotid artery stenosis, might need to increase the frequency of measurements to better follow up the progress of the risk factors.

The recorded data is collected and then stored in a secure cloud drive. Combined with personal EHRs, ML techniques are applied to provide high-precision prediction outcomes [104]. The future direction of stroke risk prediction is in developing a system including (1) various non-invasive wearables to continuously monitor the variation in risk factors; (2) customized machine learning models with high prediction power to analyze the data; (3) high precision outcomes providing dynamic short-term (weeks or days) and long-term (years) risk of stroke; and (4) a customized feedback loop for better stroke risk management. Stroke risk management includes suggestions such as living with better lifestyle behaviors, adding or increasing the frequency of certain measurements using wearables, receiving a more comprehensive or specific medical examination, or actively receiving medication or surgery to prevent stroke.

Stroke is a disease resulting from lacking blood flow in the vessels going to the brain or in the brain. However, the risk factors measured using the techniques in Stages I and II are those that trigger certain mechanisms resulting in the occurrence of stroke, which do not directly reflect the status of cerebral blood flow. Neuroimaging techniques are promising tools to characterize the status of cerebral blood flow and the related neural activity [105]. These technologies increase the value of the recordings and provide more accurate prediction in time. Often one neuroimaging technique can only monitor the brain activity in constrained frequency range at specific monitor region and depth. To increase the imaging ability, applying multimodal neuroimaging system to increase the temporal and spatial resolution becomes a trend [106,107,108,109]. Widely used neuroimaging techniques are functional MRI (fMRI), positron emission tomography (PET), CT, magnetoencephalography (MEG), TCD, photoacoustic, fNIRS and EEG [110]. However, fMRI, CT, PET, and MEG are rather costly, bulky, with low accessibility, professional operators depended for measurements and analysis as well as restricted for physical activities. These limitations impede their usability for stroke risk prediction in any of the stages mentioned in Figure 3. Among the available neuroimaging techniques, a multimodal EEG-fNIRS system is wearable, lightweight, and user-friendly for daily or bedside monitoring. Therefore, it has great potential to improve the outcome of a prediction system in Figure 3. This multi-modal system facilitates simultaneous electrical activity and hemodynamic activity recordings, which helps to study the mechanism of neurovascular coupling [107].

**Figure 4:**
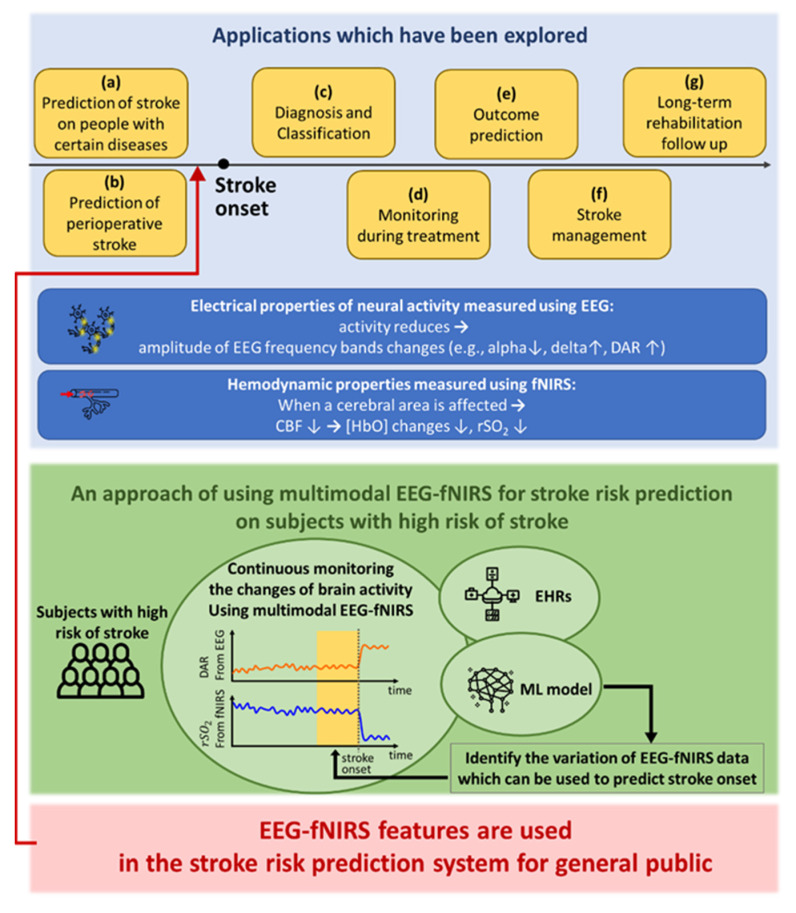
EEG or fNIRS for stroke-related applications and future perspective of applying multimodal EEG-fNIRS technique for stroke risk prediction for general public. CBF: cerebral blood flow; DAR: delta to alpha ratio; EEG: electroencephalography; EHRs: electronic health records; fNIRS: functional near-infrared spectroscopy; [HbO]: concentration of oxygenated hemoglobin; ML: machine learning; rSO_2_: regional cerebral tissue oxygenation.

The use of EEG or fNIRS neuroimaging technique on stroke-related applications are categorized in Figure 4. Examples of each application are introduced. (a) Hemodynamic monitoring on patients with migraine using fNIRS shows that vasoconstriction increases the risk of stroke [108]. (b) Combining EEG, fNIRS, and HRV is proposed to predict cerebral ischemia on patients receiving carotid endarterectomy [109]. (c) A decrease in hemoglobin oxygen saturation (StO_2_) as well as higher [HbDeoxy] distinguish patients with large vessel occlusion stroke from healthy subjects [111]. (d) EEG or fNIRS is applied to monitor the brain activity of patients receiving tissue plasminogen activator or mechanical thrombectomy prior, during and after the treatments [112,113]. (e) The changes of EEG beta, delta and theta bands are the predictors of a malignant course on acute ischemic stroke patients [114]. (f) During stroke management, EEG is used to monitor early poststroke seizures which happen in around 5% of patients two to three days after receiving thrombolysis and thrombectomy therapies [115]. (g) For rehabilitation, multimodal EEG-fNIRS is promising to define the neurovascular coupling during robot-assisted gait training [116].

### 5.2. EEG-fNIRS Multimodal Recording for Stroke Risk Prediction

The variations in neurovascular coupling during and after stroke are well studied using EEG or fNIRS as discussed in the previous section. It is concluded that Delta band power, DAR of EEG signals decrease when recanalization succeeds. Regarding fNIRS signals, [HbOxy] and rSO_2_ both increase when the blood flow is restored. In addition, the interhemispheric difference of both EEG (BSI) and fNIRS (IHΔrSO_2_) decreases when the blood clot is removed. Understanding the mechanisms of the variations of recorded signals at various stages of stroke promotes continuous EEG-fNIRS monitoring for stroke risk prediction. This approach can be conducted as follows (Figure 4).

An EEG-fNIRS system with a user-friendly cap facilitates continuous monitoring of gradual changes of cerebral blood flow and electrical brain activity due to atherosclerosis, which cannot be easily achieved using TCD headbands, CT, or MRI. To increase the willingness and benefit of the subjects wearing the EEG-fNIRS cap at least twice a day, the cap can first be applied on a cohort of subjects with a high risk of ischemic stroke for the proof-of-concept stage. According to the suggestion of applying various techniques at different stages (Figure 3), the subjects stratified into a higher risk category according to ML analysis results based on the self-reported survey, the simple wearable devices, and EHRs should be invited to receive a series of 30 min EEG-fNIRS monitoring twice a day.

It is assumed that the probability of stroke onset on this cohort would be high (people with high BP have at least five times more incidence of stroke than those with normal BP [117,118]) and EEG-fNIRS monitoring twice a day tracks the variations in brain signals during the progression of atherosclerosis, which can result in thrombotic stroke (accounts for 20% of ischemic stroke) [119]. Combining the real-time recorded data with other measured physiological parameters and EHRs allows tracking the brain activity before stroke onset in a more comprehensive overview. Further, ML algorithms will be applied to identify the key features of stroke onset. These parameters and conditions can be used in a stroke risk prediction system for the public.

### 5.3. Challenges and Limitations of EEG-fNIRS Multimodal Recording and Possible Solutions

While multi-modal EEG-fNIRS are used in many applications introduced in Section 5.1, the limitations of applying the multimodal system for brain activity monitoring and the corresponding solutions are listed in Table 4. Please be noted that the limitations of fNIRS mentioned in the table means that of continuous-wave NIRS, the most common type of NIRS used in research and available on the market.

Besides the challenges of the multimodal EEG-fNIRS system, to collect useful recordings for a stroke risk prediction system proposed in Section 5.2, the protocol of EEG-fNIRS measurements needs to be designed in detail for the benefits of test subjects and researchers. The following issues need to be explored for an optimal protocol: first, the number of EEG electrodes and fNIRS optodes of the system to be applied during measurements; more electrodes and optodes result in longer preparation time, too few electrodes and optodes can miss meaningful signals. The second issue is the cohort to recruit test subjects and the number of subjects to recruit. We would suggest recruiting subjects from communities or companies based on the stroke stratification according to their annual checkup results. The third issue is the frequency, the duration, and the moments of the EEG-fNIRS recordings, unlike wrist-worn wearables, which are user-friendly and can be worn 24 h. We suggested a series of 30 min EEG-fNIRS measurements twice a day because this may be an acceptable frequency and duration for most people. Regarding the time for the measurements, a measurement taken two hours after waking is suggested since this is the time most strokes happen [128]. Fourth, a user-friendly EEG-fNIRS system is needed to be easily worn. In the meanwhile, the accuracy and correctness of the recorded data need to be easily confirmed by the software along with the system. Fifth, after the data is obtained, the suitable machine learning models need to be studied for an optimal prediction system.

## 6. Conclusions

This paper presents the trends and challenges of wearable multimodal technologies for stroke risk prediction. We introduced and compared the available wearable devices to monitor the risk factors of stroke. To achieve an optimal prediction system providing both short-term (weeks or days) and long-term (years) dynamic risk of stroke for better stroke risk management, the system must include as many types of health data as possible, a ML model with high prediction power, and an effective feedback loop for stroke risk management. The health data includes the real-time recorded physiological parameters from the wearable devices and the EHRs. The use of a multimodal EEG-fNIRS system extents the collection domain of physiological signals and increases the prediction power of a prediction system for stroke risk. However, more efforts are needed to overcome the challenges of applying present multimodal EEG-fNIRS systems, such as increasing the spatial resolution, considering time domain- or frequency domain- NIRS to obtain the absolute values of hemoglobin species and developing a user-friendly system to maintain the user’s long-term will to wear it. With an appropriate system and an optimal protocol, the future perspective of applying EEG-fNIRS measurements on people with high risk of stroke can be a good option to investigate.

## Figures and Tables

**Table 1:** Risk factors in a scoring system specifically developed for the Chinese Population.

Risk Factors
Sex
Age
Geographic region (northern/southern China, divided by the Yangtze River)
Waist circumference
Total cholesterol
High-density lipoprotein cholesterol
Blood pressure
Antihypertensive medications within the past two weeks
Diabetes Mellitus
Current smoker
Parental history of stroke

**Table 2:** Detected risk factors and the percentage of them accounting for stroke.

Risk Factors	Percentage of Stroke-Related Risk Factor	Detection/Characterization Method
Lifestyle behaviors (combining many factors)	75%	Questionnaires
Hypertension	50%	Wearables to measure vascular related parameters
Air pollution	30%	APP on smart phone
Atrial fibrillation and abnormal electrocardiogram (ECG)	20%	Wearables to measure ECG
Carotid plaque	15%	Carotid ultrasound
Intracranial Atherosclerosis	10%	Transcranial Doppler (TCD)

**Table 3:** Comparison of computed tomography (CT), magnetic resonance imaging (MRI) (the gold standard for brain imaging) and various prediction technologies discussed in Section 3. More ‘+’ sign means the wearable technology meets the characteristic more.

	Question-Naires via Mobile APP	Mobile Phone, Air Pollution Sensor	ECG	PPG	Carotid Ultra-Sound Neckband	TCD Headband	Accelerometer + Pressure Sensors	Goggle	EMG	EEG	fNIRS	CT	MRI
Compact, light weighted	++++	++++	+++	+++	+++	++	++++	+++	+++	++	++	+	+
Low-cost of the equipment/per test	++++	++++	+++	+++	+++	++	++++	+++	+++	++	++	+	+
Accessibility	++++	++++	+++	+++	+	+	+++	++	+++	++	++	+	+
Self-service (No assistant needed)	++++	++++	++++	++++	++	+	++++	++++	++++	++	++	+	+
Frequency of test (++++: anytime, +: only when needed)	++++	++++	++++	++++	+++	+	++++	+++	++++	+++	+++	+	+
Short preparation and response time	++	++++	++++	++++	+++	+++	++++	++++	++++	+++	++++	+	+
Data continuity	+	++++	++++	++++	+++	+++	++++	++++	++++	++++	++++	+	+
High time resolution	NA	++++	++++	++	++++	+++	+++	+++	++++	++++	+++	+	+
High spatial resolution	NA	NA	+	++	++	++	++	+++	+++	++	+++	+++	++++
Broad field of view	NA	NA	++	+	+	++	+++	++	++	++++	++++	++++	++++
Often used in which stage of stroke course	Prediction	Prediction	Predic-tion	Predic-tion	Prediction	Prediction	Prediction, rehabilitation	Detec-tion	Detec-tion, rehabili-tation	Rehabilitation	Rehabilitation	Detec-tion	Detec-tion

**Table 4:** Limitations of a multimodal EEG-fNIRS system and the possible solutions.

Limitations of EEG, fNIRS or a Multimodal EEG-fNIRS System for Stroke Risk Prediction		Possible Solutions to Overcome the Limitations
EEG	Spatial resolution of 5–9 cm [120]	fNIRS with spatial resolution of 2–3 cm can be combined with EEG to increase the spatial resolution [120].
fNIRS	Poor sensitivity to the deep brain cortex, where 20% of stroke, named lacunar stroke, occurs [111]	Introduce high-density diffuse optical tomo/topography (DOT) [121]
	Signals are affected by the scalp-related hemoglobin oscillation or contamination from extra-cerebral layers	Design experiment carefully: Reduce the distance of optodes to record baseline signals and then deduct them from the other recorded signals [122].
		Improve NIRS techniques: Time domain-NIRS and Frequency domain-NIRS can separate the contribution from extra-cerebral layer or cortical part [111,121].
		Apply statistical processing: Long-term continuous monitoring minimizes the influence from region not in interests [123].
	Absolute values of [HbOxy], [HBDeoxy], [HbT = HbOxy + HbDeoxy] (∝ cerebral blood volume), StO_2_ (hemoglobin oxygen saturation) are not available, only the variation is available, so the threshold values for stroke onsets cannot be determined	Use TD-NIRS and FD-NIRS to characterize the absolute values of hemoglobin species [111,121].
	CBF cannot be perfectly measured	Diffuse correlation spectroscopy (DCS) can continuously monitor CBF index [124].
EEG-fNIRS	Difficult to record neuronal electrical and hemodynamic activity from the same location.	Sophisticated hardware developments and integration of fNIRS optodes and EEG electrodes are needed [125].
	Many optodes are needed to cover the area of interest, may lack of scalp space when applying EEG with fNIRS	
	The cap/headset may cause discomfort for long-term use	A wireless EEG-fNIRS system and a proper design, even customization, of the fixation devices are needed [126,127].

## Data Availability

Data sharing not applicable.
